# Atrial Fibrillation Detection on the Embedded Edge: Energy-Efficient Inference on a Low-Power Microcontroller

**DOI:** 10.3390/s25216601

**Published:** 2025-10-27

**Authors:** Yash Akbari, Ningrong Lei, Nilesh Patel, Yonghong Peng, Oliver Faust

**Affiliations:** 1 School of Computing and Information Science, Anglia Ruskin University Cambridge Campus, East Rd., Cambridge CB1 1PT, UK; yash.akbari@hotmail.com (Y.A.); yonghong.peng@aru.ac.uk (Y.P.); 2 School of Engineering and Built Environment, Sheffield Hallam University, Howard Street, Sheffield S1 1WB, UK; n.lei@shu.ac.uk; 3 Rhythm & Retina Clinic, 401, Rhythm House, Gotalawadi Rd., Lal Darwaja, Surat 395003, Gujarat, India; drnileshparshottam@gmail.com

**Keywords:** atrial fibrillation, real time, energy consumption, embedded

## Abstract

Atrial Fibrillation (AF) is a common yet often undiagnosed cardiac arrhythmia with serious clinical consequences, including increased risk of stroke, heart failure, and mortality. In this work, we present a novel Embedded Edge system performing real-time AF detection on a low-power Microcontroller Unit (MCU). Rather than relying on full Electrocardiogram (ECG) waveforms or cloud-based analytics, our method extracts Heart Rate Variability (HRV) features from RR-Interval (RRI) and performs classification using a compact Long Short-Term Memory (LSTM) model optimized for embedded deployment. We achieved an overall classification accuracy of 98.46% while maintaining a minimal resource footprint: inference on the target MCU completes in 143 ± 0 ms and consumes 3532 ± 6 μJ per inference. This low power consumption for local inference makes it feasible to strategically keep wireless communication OFF, activating it only to transmit an alert upon AF detection, thereby reinforcing privacy and enabling long-term battery life. Our results demonstrate the feasibility of performing clinically meaningful AF monitoring directly on constrained edge devices, enabling energy-efficient, privacy-preserving, and scalable screening outside traditional clinical settings. This work contributes to the growing field of personalised and decentralised cardiac care, showing that Artificial Intelligence (AI)-driven diagnostics can be both technically practical and clinically relevant when implemented at the edge.

## 1. Introduction

Atrial Fibrillation (AF) is the most prevalent sustained cardiac arrhythmia, affecting an estimated 59 million individuals globally as of 2019 [1]. It is characterised by rapid and disorganised atrial electrical activity that disrupts coordinated atrial contraction, thereby promoting the stasis of blood flow, particularly in the left atrial appendage. This contributes to a substantially elevated risk of thromboembolic events, most notably ischemic stroke, for which AF increases the risk fivefold [2,3]. Moreover, AF is associated with a broader spectrum of adverse clinical outcomes, including heart failure [4], cognitive impairment [5], reduced quality of life [6], and increased all-cause mortality [7,8]. Despite its clinical significance, timely AF diagnosis remains challenging. A considerable proportion of AF cases are asymptomatic or manifest paroxysmally; hence, such cases are difficult to detect during routine clinical encounters [9]. This delay is particularly problematic, as early initiation of evidence-based treatments, such as anticoagulation therapy, rate or rhythm control, and lifestyle changes, has been shown to significantly reduce the morbidity and mortality [10]. These challenges underscore the imperative for continuous, accessible, and reliable AF monitoring modalities that extend beyond conventional clinical settings.

Remote monitoring technologies are seen by many as promising tools for improving AF detection rates, particularly for paroxysmal and silent AF [11,12,13]. Wearable devices and portable biosensors facilitate the longitudinal acquisition of physiological data in real-world environments, enabling proactive intervention and management. However, many current solutions rely on cloud-based analytics [14], which introduce several critical limitations: increased latency, dependence on uninterrupted wireless connectivity, elevated energy consumption due to frequent data transmission, and concerns related to data privacy and regulatory compliance [15]. To mitigate these constraints, a shift toward edge computing is necessary. The “edge” is often defined as a continuum of processing locations; while the network edge refers to computing near the base station, our focus is on the embedded edge or micro edge: the processing power located directly on the end-device (the wearable sensor). Deploying edge Artificial Intelligence (AI) models at this tier allows for real-time signal processing, drastically reduces energy expenditure by minimising raw data transmission, and bolsters user privacy through localised inference on resource-constrained Microcontroller Units (MCUs) [16,17].

In this work, we propose a novel embedded edge AF detection system that performs real-time classification entirely on a low-power embedded platform. Our approach represents a classic TinyML application, where the complex model training occurs in the cloud, but the resultant lightweight model is deployed to the MCU for local execution. Our method is based on Heart Rate Variability (HRV) features derived from RR-Intervals (RRIs) as the primary input, rather than raw Electrocardiogram (ECG) waveforms. We demonstrate that this abstraction significantly reduces the computational burden and memory footprint while retaining sufficient discriminatory power for arrhythmia detection. The demo setup is based on the design and implementation of a compact Long Short-Term Memory (LSTM) model, which was optimised for embedded deployment. The model achieved a classification Accuracy (ACC) of 98.46%. Critically, we quantified the resource footprint of this architecture: inference on the selected MCU completes in approximately 143 ± 0 ms and consumes an average current of 12.85 mA at 1.8 V, corresponding to an energy consumption of 3532 ± 6 μJ per inference. This low energy cost for inference enables a key architectural advantage: the wireless uplink can be strategically kept OFF during continuous monitoring and activated only to transmit a high-priority alert upon AF detection, thereby conserving energy and maximising the battery life. We validate the proposed architecture through detailed empirical measurements on a representative MCU platform, demonstrating the feasibility of sustainable continuous AF monitoring via intelligent edge processing. This contribution advances the development of energy-efficient, privacy-preserving, and clinically meaningful wearable diagnostics for cardiac rhythm disorders. It also aligns with emerging trends in personalised and decentralised healthcare, where AI-driven insights are delivered directly to the user with minimal latency and infrastructural demands.

The manuscript is structured as follows: The Section 2 introduces the materials and methods used to implement and validate the LSTM model on an MCU. Section 2.5 includes a system architecture sketch clarifying the roles of the MCU (micro edge), the wireless link, and the cloud. We also outline the measurement setup which allows us to establish the power requirements during inference. The Section 3 details the model performance results and the implementation related measurements. In the Section 4, we compare and contrast these results with reported performance from the related work. The paper concludes with Section 5.

## 2. Materials and Methods

**Figure 1:**
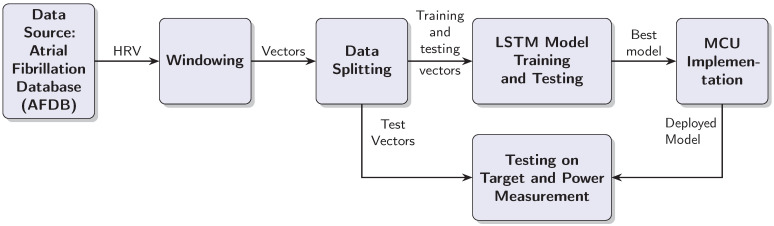
Block diagram of the AF detection system development and deployment process.

This section outlines the steps taken to test the LSTM based AF detection model with an MCU target. The design process is iterative, because the MCU target places restrictions on the model complexity and the window length. Furthermore, the practical implementation should be capable of processing RRIs in real time, which means processing a test vector in less than half a second. As a consequence of these requirements, we had to balance the classification performance on one side with the computational complexity of the model and inference time on the other. The design process resulted in a linear data processing chain, which is documented in Figure 1. The individual blocks are explained in the text below.

### 2.1. Data Source

The AF detection model was developed with HRV data from the MIT-BIH AF Database [18,19]. The collection comprises 23 ten-hour, two-lead ECG Holter recordings (sampling rate = 250 Hz) recorded from distinct subjects. Expert cardiologists, from Beth Israel Hospital, Boston, MA, United States, provided beat-level and rhythm-level annotations, including AF markers and R-peak positions. For this study, the R-peak annotations were used to derive the RRI sequence for HRV analysis.

To validate the model’s generalisation capability to unseen less-preprocessed data from a separate cohort, we utilised the Long-Term AF Database (LTAFDB) [18,20]. The LTAFDB consists of 84 long-term ECG recordings (typically 24 to 25 h each) from subjects with paroxysmal or sustained AF. The beat and rhythm annotations used for generating the RRI vectors were carried out by medical experts from the Northwestern University, 633 Clark St, Evanston, IL 60208, USA.

### 2.2. Windowing

**Figure 2:**
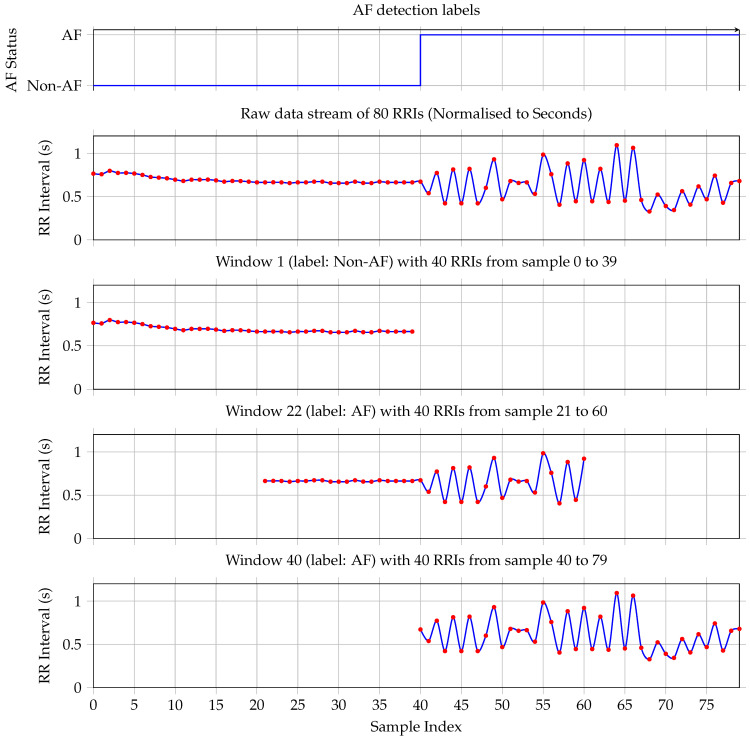
The first plot shows the ground truth—each RRI is labeled as either non-AF or AF. The plot shows a transition from non-AF to AF on sample RRI sample 40. The second plot shows the corresponding RRI stream. Window 1 contains only non-AF samples. Therefore, the label for the whole window is non-AF. Plot four shows Window 22, which is taken at the transition from non-AF to AF. It has more RRIs labeled as AF; hence, the complete window is labeled as AF. Plot five documents window 40, which contains only RRIs labeled as AF. Hence, window 40 is labeled as AF.

Data from 18 subjects were used for model development and evaluation. Preprocessing started with the segmentation of RRI sequences using a sliding window approach, as illustrated in Figure 2. Two parameters govern this process: a fixed window length of 40 RRIs and a step size of 1, resulting in a one-step sliding window technique that generates overlapping training and testing samples. Appendix A provides a short discussion and justification for selecting a window length of 40 RRIs.

Each window is assigned a binary label based on the proportion of AF beats it contains. Specifically, if a given window includes 20 or more AF-labeled beats (i.e., at least 50% of the window), it is labeled as AF; otherwise, it is labeled as non-AF. This threshold is selected to align with the minimum clinical duration criteria for AF diagnosis. Given a typical heart rate, a 40-RRI window approximates the 30 s minimum duration required for an AF episode to be considered clinically significant [6,19]. The 50% threshold ensures balanced sensitivity to paroxysmal onset/offset while maintaining training robustness.

To illustrate this procedure, consider the 80 RRIs shown in the second plot of Figure 2. These intervals yield 41 overlapping windows. The first window, comprising the first 40 RRIs, is depicted in the second plot and it contains no AF-labeled beats; hence, it is classified as non-AF, consistent with the ground truth shown in the first subplot. The fourth plot illustrates the first window that meets the AF threshold: it contains exactly 20 AF beats and is therefore labeled as AF. Finally, the last subplot shows the 41st window, which consists entirely of AF-labeled RRIs and is likewise labeled as AF.

This windowing strategy ensures dense temporal coverage and supports the robust training of sequence-based classifiers by preserving the beat-wise temporal structure while allowing for smooth transitions between AF and non-AF states.

### 2.3. Data Splitting

The windowing process resulted in set of 1,127,641 labeled RRI vectors. The dataset was randomly partitioned into two disjoint subsets, with 80% of the data (ł845,730) allocated for training, and the remaining 20% (281,911) were used for testing.

### 2.4. Model Training and Testing

Our AF detection system utilises a Bidirectional-LSTM model [21], the architecture of which is detailed in Table 1. The model is designed to process the 40-sample RRI input vectors. The core of the model consists of two parallel LSTM layers (Layers 2a and 2b), operating in forward and backward directions, respectively, each producing an output of 40 features. These recurrent layers are followed by a Global 1D Max Pooling layer (Layer 3) to reduce the dimensionality and extract the significant features. A fully connected layer with ReLU activation (Layer 4) then processes these features. The subsequent Dropout layer (Layer 5) was included for regularisation, in order to prevent overfitting during training. The final output is generated by a sigmoid-activated fully connected layer (Layer 6), yielding a single probability score for AF detection. The total number of trainable parameters for the model is 17,561.

The model was developed in Python (v3.12) using the Keras Application Programming Interface (API) for the TensorFlow network (v2.20.0). The model was trained using the designated training vector set. During training, the model’s parameters were optimised to minimise a binary cross-entropy loss function, using the Adam optimiser [22]. Early stopping was implemented to prevent overfitting. Upon completion of training, the model with the best performance on the validation set was selected for implementation testing on the MCU target and for external validation on the LTAFDB (see Section 3.2.2).

Our empirical analysis (Section 3.2.2 and Section 3.2.3) focuses on quantifying the inference energy on the embedded edge (MCU), validating the feasibility of this power-saving energy-gated system design.

### 2.5. MCU Implementation

For the practical implementation of the AF detection model, we used the STM32L475VGT6 device [23], which is an ultra-low-power microcontroller based on the Arm Cortex-M4 32-bit RISC core [24] operating at a frequency of up to 80 MHz [25]. The Cortex-M4 core features a floating point unit single precision, which supports all Arm single-precision data-processing instructions and data types. It also implements a full set of Digital Signal Processing (DSP) instructions and a memory protection unit, which enhances application security. The device has 1 Mbyte flash memory and 128 kByte of Static Random Access Memory (SRAM).

### 2.6. Testing on Target and Power Measurement

**Figure 3:**
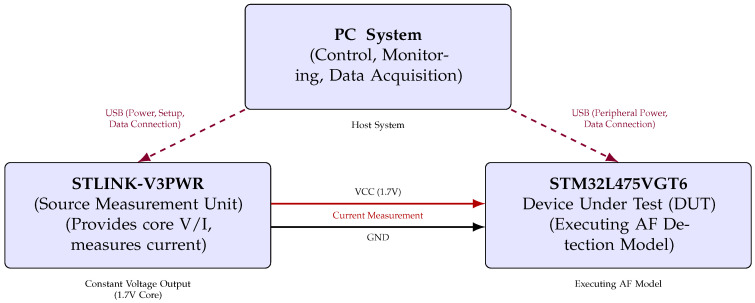
Functional block diagram of hardware setup for STM32L475VGT6 AF detection model energy measurement (triangular layout).

To evaluate the model’s performance on the target hardware and to quantify its energy as well as its latency characteristics during inference, a dedicated testbench was constructed. This setup is composed from three primary components: a host Personal Computer (PC) system, the STM32L475VGT6 MCU serving as the Device Under Test (DUT), and the STLINK-V3PWR source measurement unit, as illustrated in Figure 3.

The host PC served as the central controller, managing test execution through a bidirectional serial interface with the MCU. It also triggered and synchronised power measurements via a dedicated USB connection to the STLINK-V3PWR module. The DUT processor was powered via the source measurement unit, which provided a constant supply voltage of 1.7 V to the STM32L475VGT6 and simultaneously measured the current drawn during inference.

To ensure the reliability and statistical robustness of our energy and timing metrics, the measurements were performed through a set of repeated runs. Specifically, we conducted 30 independent power measurement trials (*N* = 30). In each trial, the MCU performed 20 consecutive model inferences. The inference time, power consumption, and energy per inference were recorded for each trial. This methodology allowed us to establish the mean and variance of the key parameters, providing a statistically sound measure of the system’s performance.

Each test run was initiated by triggering the power measurement sequence on the STLINK-V3PWR, followed by the activation of model inference on the MCU. This controlled process enabled the precise measurement of the inference latency and the instantaneous power consumption. The STM32 device executed the AF detection model entirely on-chip, allowing assessment under realistic embedded deployment conditions.

The triangular layout in Figure 3 depicts the connections between the components, highlighting the power delivery, current sensing paths, and data communication channels. This configuration ensured high temporal resolution in power measurement, critical for accurately quantifying the energy efficiency of edge-based AI inference.

## 3. Results

### 3.1. Baseline Performance on PC

**Figure 4:**
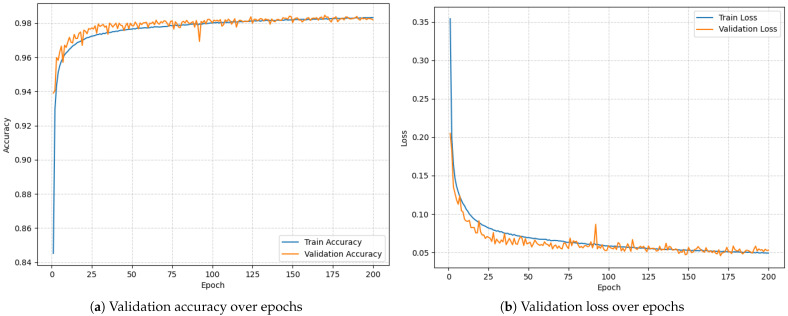
Model training over 200 epochs.

To establish a performance benchmark, the LSTM model was initially trained and evaluated on a PC using preprocessed RRI data. The model was trained for 200 epochs using a categorical cross-entropy loss function. Figure 4 documents the training process. The final validation results were as follows:

- Validation Accuracy: 98.46%;
- Validation Loss: 0.0456.

These results confirm that the model demonstrates strong discriminative ability for AF detection under ideal (non-constrained) computing conditions, providing a robust reference point for subsequent evaluation on embedded hardware.

### 3.2. Embedded Performance on STM32 Microcontroller

The trained model was converted using the X-Cube-AI framework and deployed to the STM32L475VGT6 microcontroller for real-time inference. The same validation dataset was used to compare performance metrics between the PC and embedded platforms.

#### 3.2.1. Confusion Matrix Analysis

**Figure 5:**
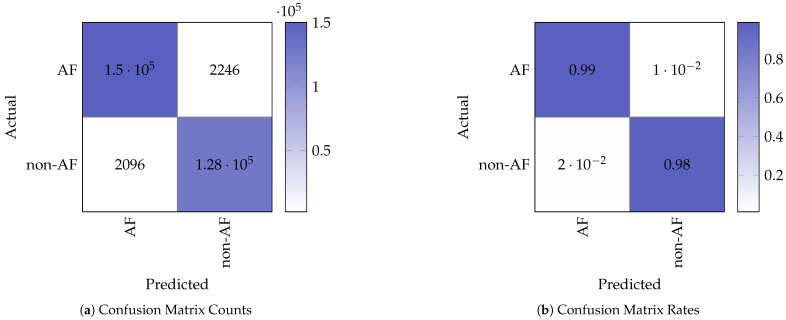
Confusion matrix representation of the model test results.

Figure 5 shows the confusion matrix counts and corresponding normalised rates obtained from testing on the STM32. The confusion matrix indicates the following performance values for the binary (non-AF and AF) classification:

- True Positives (TP): 127,589;
- False Negatives (FN): 2096;
- False Positives (FP): 2246;
- True Negatives (TN): 149,980.

From the performance values, we computed the following performance metrics:

- Accuracy, see Equation (1).
  (1) $$\frac{TP+TN}{TP+TN+FP+FN}=\frac{127589+149980}{281911}=98.46\%$$
- Sensitivity (Recall), see Equation (2).
  (2) $$\frac{TP}{TP+FN}=\frac{127589}{127589+2096}=98.38\%$$
- Specificity, see Equation (3).
  (3) $$\frac{TN}{TN+FP}=\frac{149980}{149980+2246}=98.52\%$$
- Precision (Positive Predictive Value), see Equation (4).
  (4) $$\frac{TP}{TP+FP}=\frac{127589}{127589+2246}=98.27\%$$
- F1-score, see Equation (5).
  (5) $$2\times \frac{\mathrm{Precision}\times \mathrm{Recall}}{\mathrm{Precision}+\mathrm{Recall}}=2\times \frac{0.9827\times 0.9838}{0.9827+0.9838}=98.33\%$$

These metrics demonstrate that the model retains strong classification performance when deployed on the embedded device, with minimal degradation in AF detection performance compared to the baseline model running on the PC.

#### 3.2.2. External Validation with the LTAFDB

To assess the model’s generalisation capability on a fully independent dataset, we tested the trained model on 8400 random vectors from the LTAFDB (100 vectors from each of the 84 subjects). The performance on this external, unseen dataset was as follows:

- Accuracy: 94.44%;
- Evaluation Loss (L_*eval*_): 0.2495.

The accuracy of 94.44% on the LTAFDB confirms the model’s generalisation to a different less-curated patient cohort than the one used for training. The evaluation loss is reported here as a standard metric to quantify the distance between the model’s predicted probabilities and the ground truth labels, showing the confidence and fidelity of the model’s predictions on the external data.

#### 3.2.3. Inference Time and Power Efficiency

Real-time inference timing was measured using the on-board TIM16 hardware timer. The model inference time for a window of 40 RRIs was approximately 143 ± 0 ms per sample. This corresponds to a theoretical maximum of ≈7 inferences per second, or ≈419 inferences per minute. This is well beyond the requirements for real-time AF monitoring, which is one inference for each heartbeat.

### 3.3. Power and Energy Consumption

A key objective of this work was to develop the project into a wearable low-power system able to operate continuously over a long time scale. In order to guarantee that the model can be used efficiently without the continuous need to recharge it, the power consumption was tracked at various stages in the system.

**Figure 6:**
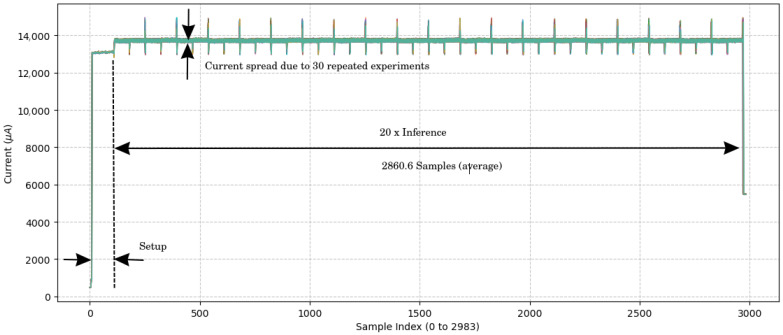
Current measurement over time samples. The figure results from an overlay of 30 experiment results. The sample period is 1 ms, which results from a 1 kHz sampling frequency for the current measurement. No variance in the inference time was observed.

Power consumption was estimated using an ST Link V3PWR [26] at the time of inference. Figure 6 documents the measurement resutls. The amperage current during inference was 13.72 ± 0.02 mA at a voltage of 1.8 V. The average current times the voltage results in the average power consumption: 13.72 ± 0.02 mA × 1.8 V = 24.70 ± 0.04 mW. This low power consumption saves battery and enables prolonged operation; hence, it is ideal for a wearable device. Given the inference time of 143 ± 0 ms, the energy consumption is the power required during the inference: 24.70 ± 0.04 mW × 143 ± 0 ms = 3532 ± 6 μJ.

## 4. Discussion

This study demonstrates the successful design, implementation, and validation of an LSTM-based AF detection system on a resource-constrained microcontroller. The primary contribution lies not in achieving the absolute highest classification accuracy reported in the literature but in architecting a solution that is practical, energy-efficient, and suitable for continuous real-world monitoring. Our approach uses HRV features to perform all inference directly on the edge, addressing the critical limitations of cloud-dependent systems, namely latency, connectivity, power consumption, and data privacy. The results, an accuracy of 98.46%, a 143 ± 0 ms inference time, and a power consumption of 24.70 ± 0.04 mW, balance clinical utility and technical feasibility for wearable diagnostic devices.

### 4.1. Architectural Context: Realising AF Monitoring on the Embedded Edge

The core contribution of this work lies in demonstrating the energy efficiency of AF inference directly on a resource-constrained MCU, a paradigm known as embedded edge AI or TinyML. This approach allows for a practical sustainable system architecture, which we clarify here to define our use of “edge” and the intended role of the entire system.

**Figure 7:**
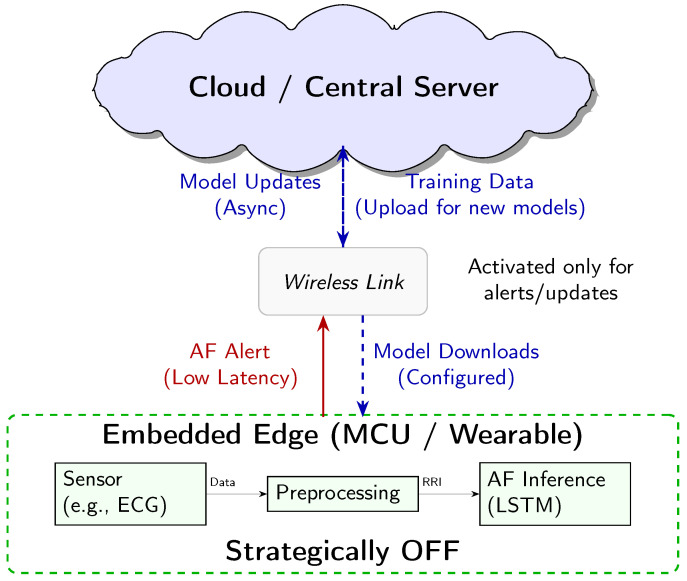
Conceptual three-tier system architecture for AF detection. The embedded edge MCU performs continuous low-power inference. The Cloud handles heavy-duty tasks like model training and storage. The Wireless link acts as a communication gate, only activated for low-latency alerts and asynchronous model updates.

The “edge” is interpreted here as the micro edge, the end device itself. Our system is designed for an energy-optimised three-tiered architecture (Figure 7), where each component has distinct power-managed roles:

1. Embedded Edge (Low-Power MCU): This tier, the focus of our empirical analysis, performs continuous, real-time AF classification. The measured energy consumption of 3532 ± 6 μJ per inference is the key enabling factor for the overall system’s efficiency.
2. Cloud/Central Server (Training and Updates): This high-resource environment is used for computationally intensive tasks, namely LSTM model training and long-term data storage. The finalised model is deployed to the MCU, and model updates are delivered asynchronously.
3. Wireless Link (Energy-Gated Uplink): The role of the wireless link (e.g., Bluetooth Low Energy) is strictly minimised to conserve power. Data transmission is the single largest energy sink in a wearable system. Therefore, the link is designed to be kept OFF during routine monitoring and only activated upon positive AF detection by the MCU. This enables the transmission of a time-stamped low-latency alert packet, drastically reducing the communication overhead.

Crucially, while our empirical measurements focused on quantifying the energy cost of the embedded edge inference engine, this three-tier model represents the necessary clinically relevant deployment scenario, enabled by our highly efficient local processing. This design minimises the latency for critical diagnosis, while maximising the energy conservation and user privacy by keeping the raw physiological data localised.

### 4.2. Preprocessing and Real-World Applicability

The feasibility of any wearable AI system depends on the entire data chain, including sensor based data acquisition and preprocessing (noise filtering, R-peak detection), as shown in Figure 7. These processing steps are known sources of computational burden and vulnerability to artifacts (e.g., motion). However, the primary scope of this manuscript is centered on demonstrating the technical and energy-cost viability of the AI inference engine itself. Specifically, we isolate and quantify the resource usage of the LSTM model deployed on a resource-constrained MCU, which represents the most computationally demanding and novel component of the system.

Our design choice is based on the pragmatic assumption that our solution will be integrated as an intelligent decision support module within existing or commercially mature HRV monitoring platforms. In such a scenario, we offer the following:

1. Decoupling the Front-End: we treat the input to our model as the preprocessed RRI sequence, effectively offloading the well-understood tasks of signal capture, noise reduction, and R-peak detection to the existing front-end sensor solution.
2. Integration Potential: this approach means our energy-efficient AF classification module can be “dropped into” an existing HRV data pipeline, simplifying the challenge for system integrators and providing a core technological advancement with minimal additional computational or energy burden.

Therefore, while the full sensor-to-cloud chain is essential for a final commercial product, a detailed hardware-specific analysis is beyond the scope of this paper, which focuses on AI optimisation and embedded deployment. Our aim is to provide a foundational assessment of our AI system’s feasibility, which is the necessary step before committing to expensive and hardware-specific full-system integration.

### 4.3. Performance in the Context of Practical Application

**Figure 8:**
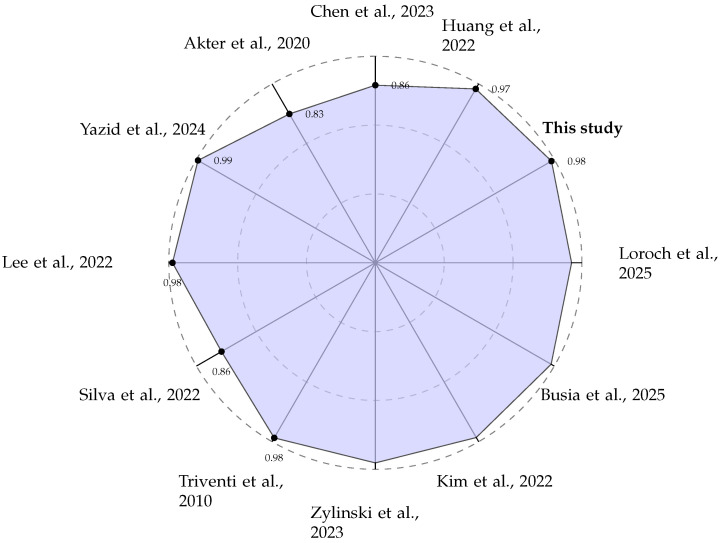
Radar plot comparing the reported classification ACC (in %) of various edge-based AF detection systems [27,28,29,30,31,32,33,34,35,36,38].

A direct comparison of performance metrics, as summarised in Table 2 and visualised in Figure 8, shows that our system is competitive with other state-of-the-art edge AF detection methods. While some studies, such as Yazid et al. [27], report slightly higher accuracy (99.13%), it is crucial to analyse the methodologies used to generate these results. We adopted a sliding window approach for creating training and testing data segments, as documented in Section 2.2. This method is intentionally designed to mimic a real-world scenario where the device has no prior knowledge of the underlying cardiac rhythm. The window moves continuously along the signal, creating data segments that may contain purely normal beats, purely AF beats, or, critically, a mixture of both, particularly at the onset or termination of a paroxysmal AF episode.

This approach contrasts sharply with methodologies that use “clean” or pre-selected data segments. For instance, Yazid et al. [27] explicitly state that segments containing both AF and non-AF beats were discarded. Similarly, other high-performing models often rely on datasets where the signal windows are curated to contain only one type of rhythm [29]. While this preprocessing simplifies the classification task and can artificially inflate the reported accuracy, it does not reflect the challenges of continuous unsupervised monitoring. A model trained on these ’pure’ vectors may perform poorly when faced with the transitional or ambiguous signal patterns common in a clinical setting. Therefore, the ACC we achieved of 98.46%, was established with a more challenging and realistic test set. Arguably this represents a more reliable indicator of the system’s practical utility.

Furthermore, the performance cannot be assessed on the accuracy alone. For an edge device intended for long-term use, inference time and the energy-accuracy trade-off are important parameters as well. Our system achieves a rapid 143 ± 0 ms inference time, enabling near-real-time feedback. More importantly, the power draw of 24.70 mW (translating to just 3532 ± 6 μJ per inference) is significantly lower than that reported by Yazid et al. [27] (89.1 mW), indicating superior energy efficiency and the potential for longer battery life in a wearable form factor.

This 3532 ± 6 μJ per inference result positions our chosen LSTM architecture as a benchmark point on the energy–accuracy landscape (the Pareto front). While we did not perform a full hyperparameter search across model sizes (e.g., 32 to 128 hidden units) to determine the absolute global optimum, the comparison in Table 2 demonstrates a superior energy-to-accuracy ratio in the context of the published edge implementations. Our software-based solution on a general-purpose MCU was deliberately chosen for its balance of performance, cost, and adaptability. This stands in contrast to System on Chip (SoC) methodologies where AI models are implemented directly in logic circuits [39,40,41,42]. While these hardware implementations can yield extremely fast inference times, a critical trade-off has to be resolved. If implemented in fixed silicon (ASICs), the model functionality becomes permanent, preventing updates or improvements without a costly hardware redesign. Alternatively, reconfigurable hardware like Field Programmable Gate Arrays (FPGAs) allows for model updates [40], but these devices are typically more expensive and power-hungry when compared to general-purpose MCUs. Our approach prioritises flexibility and low power, making it a more practical choice for scalable and maintainable wearable health solutions.

### 4.4. Limitations and Future Work

Despite the promising results, this study has several limitations that provide clear directions for future research. Our validation was performed on a well-established public dataset but did not employ a k-fold cross-validation scheme. Future work should incorporate a 10-fold cross-validation methodology to ensure the model’s robustness and to provide a more comprehensive assessment of its generalisation capabilities. Moreover, testing the system on more diverse multi-center datasets, including those with a wider range of signal qualities and patient demographics, will also be required to shows its clinical readiness.

The current implementation runs on a general-purpose MCU without a dedicated neural processing unit. While this demonstrates the model’s efficiency on common hardware, future iterations could explore deployment on MCUs equipped with AI accelerators. Such hardware could further reduce the inference time and power consumption, potentially enabling the use of more complex models for enhanced diagnostic accuracy or the classification of multiple arrhythmia types without compromising battery life.

A significant avenue for future enhancement is the incorporation of Explainable AI (XAI) [43]. For any diagnostic support tool to gain clinical acceptance, it must not be a “black box”. Clinicians need to trust and understand the basis of an AI-driven recommendation. Future research should focus on implementing lightweight XAI techniques, such as Local Interpretable Model-agnostic Explanations (LIME) or Shapley Additive Explanations (SHAP) adapted for embedded systems. These methods could highlight which specific HRV features or beat-to-beat intervals were most influential in a given AF classification, providing valuable interpretable feedback that could aid in clinical decision-making.

Another promising direction is the expansion towards multimodal signal analysis. While HRV is a powerful indicator for AF, its specificity can be limited by artifacts or other arrhythmias. Future systems could integrate data from other sensors, such as Photoplethysmogram (PPG) or accelerometers, as proposed by emerging platforms like BioBoard [44]. Fusing data from multiple sources could create a more robust system capable of distinguishing true AF from motion artifacts and potentially classifying other conditions, thereby increasing the overall diagnostic value of the wearable device.

Finally, we envision the evolution of such edge devices within a broader healthcare ecosystem [45]. The role of AI at the edge of the network is not necessarily to provide a definitive diagnosis, which is still left to clinicians, but to act as an intelligent gatekeeper or triage system. Therefore, the primary goal is to maximise sensitivity, which ensures that no potential AF event is missed. The requirement for specificity can be relaxed, as a suspected event can trigger an alert for the user to seek clinical confirmation, or the device can automatically transmit a short relevant ECG snippet to a healthcare provider. This approach aligns with the goal of edge computing: minimising data transmission and power consumption by only communicating and acting upon data that are deemed significant. This “high-sensitivity” screening model represents the most pragmatic and impactful application of AF detection on the edge.

## 5. Conclusions

This study presents a practical and energy-efficient approach to AF detection using a lightweight LSTM model deployed on a resource-constrained MCU. Rather than focusing solely on achieving the highest theoretical accuracy, we have prioritised a design that balances clinical utility, real-time responsiveness, and power efficiency—key requirements for continuous monitoring in wearable devices.

By using HRV features and optimising the model for Embedded Edge inference, we demonstrate that high accuracy (98.46%) and low latency (143 ± 0 ms) are achievable. Crucially, the ultra-low energy consumption of 3532 ± 6 μJ per inference validates our proposed energy-gated architectural strategy. This approach minimises energy expenditure by enabling the device to perform continuous AF screening locally and strategically keeping the wireless communication link OFF, activating it only to transmit a critical alert. This effectively bypasses the high-power, latency, and privacy issues associated with cloud offloading.

Our findings show that real-world conditions, such as signal transitions and mixed-rhythm segments, can be effectively handled by the proposed system, making it robust to practical deployment challenges. The solution stands out for its adaptability, scalability, and ability to operate on general-purpose MCUs, avoiding the rigidity and cost associated with fixed-function hardware accelerators.

This work affirms the viability of performing clinically relevant AF detection at the micro edge. It offers a blueprint for developing intelligent wearable cardiac monitoring solutions that are not only technically feasible but also designed for long-term energy-efficient operation in real-world environments. By achieving this high standard of embedded edge AI performance, we have laid the foundation for scalable personalised cardiovascular care, enabling intelligent screening systems to proactively support patients and clinicians in both home and clinical settings.

## Figures and Tables

**Table 1:** Layer-wise architecture for the 40 × 64 bidirectional-LSTM model.

Layer	Type	Output Shape	Number of Parameters
1	Input	40, 1	0
2a	LSTM (forward)	40, 40	6720
2b	LSTM (backward)	40, 40	6720
3	Global 1D max pooling	80	0
4	Fully connected ReLU	50	4050
5	Dropout	50	0
6	Fully connected sigmoid	1	51

**Table 2:** Performance comparison with state-of-the-art MCU based edge AI systems for AF detection.

Author, Year	Data	Method	Performance
Triventi et al., 2010 [28]	Clinical data	Mahalanobis distance classifier	ACC = 97.9%
Lee et al., 2022 [29]	ECG-signals from Korea University Anam Hospital in Seoul	Resnet-100	ACC = 98.2%
Huang et al., 2022 [30]	Measured signals	Sorting fuzzy min–max	ACC = 97.21%
Silva et al., 2022 [31]	PTB-XL database	Convolutional neural networks	ACC = 94.1%
Chen et al., 2023 [32]	AF Challenge	Multi-layer perceptron	ACC = 86%
Akter et al., 2024 [33]	PTB-XL ECG signal database	CNN-Bi-LSTM	ACC = 83.21%, inference time = 0.85 s
Yazid et al., 2024 [27]	MIT-BIH AF database	Support vector machine	ACC = 99.13%, inference time = 11.28 ms, power consumption ≈ 89.1 mW
Zylinski et al., 2023 [34]	PhysioNet 2020	Support vector machine	ACC = 96.9%, inference time = 720 μs
Kim et al., 2022 [35]	MIT-BIH AF database	Lightweight CNN	ACC = 97.7, time = 298 ms, current consumption ≈ 3.55 mA
Busia et al., 2025 [36]	MIT-BIH arrhythmia	Tiny transformer	ACC = 98.97%, inference time = 4.28 ms, energy consumption ≈ 0.09 mJ
Fajardo et al., 2025 [37]	Not specified	Custom CNN (CNN5)	F1 Score = 90.9%, time = 28.5 ms, current consumption ≈ 100 mA
Loroch et al., 2025 [38]	Not specified	Deep neural network	ACC = 95%, power consumption ≈ 3.8 mW
This study	MIT-BIH AF database	LSTM	ACC = 98.46%, inference time = 143 ± 0 ms, power consumption ≈ 24.70 mW.

**Figure A1:**
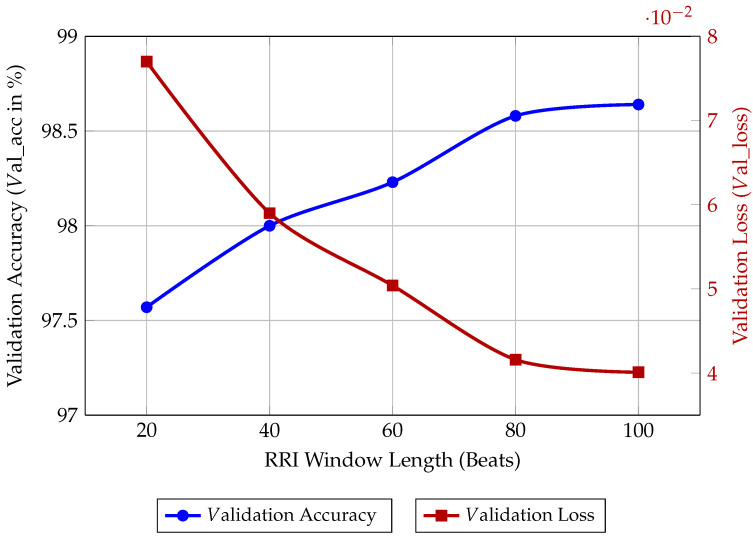
Model performance vs. RRI window length based on 40 epochs of training.

## Data Availability

The AF detection model was designed with the public ’MIT-BIH AF Database’ available at https://physionet.org/content/afdb/1.0.0/ (accessed on 20 October 2025). The AF detection model was validated with the public ’Long Term AF Database’ available at https://physionet.org/content/ltafdb/1.0.0/ (accessed on 20 October 2025).

## Abbreviations

**ACC**	Accuracy
**AF**	Atrial Fibrillation
**AFDB**	Atrial Fibrillation Database
**AI**	Artificial Intelligence
**API**	Application Programming Interface
**XAI**	Explainable AI
**DSP**	Digital Signal Processing
**DUT**	Device Under Test
**ECG**	Electrocardiogram
**FPGA**	Field Programmable Gate Array
**HRV**	Heart Rate Variability
**LIME**	Local Interpretable Model-agnostic Explanations
**LSTM**	Long Short-Term Memory
**MCU**	Microcontroller Unit
**PC**	Personal Computer
**PPG**	Photoplethysmogram
**RRI**	RR-Interval
**SHAP**	Shapley Additive Explanations
**SoC**	System on Chip
**SRAM**	Static Random Access Memory

## Appendix A. Analysis of RRI Window Length

To substantiate the selection of the 40-beat RRI window length for our Bi-LSTM model, we performed an ablation study on the input sequence length. The window length is a critical hyperparameter that directly governs the balance between diagnostic performance, memory footprint, and computational cost on an MCU.

We trained and evaluated the fixed 40 × 64 Bi-LSTM architecture using five different window lengths (L = 20, 40, 60, 80, and 100 beats) for 50 epochs each. The results, presented in Table A1 and visualised in Figure A1, illustrate the following trade-offs:

- Performance Saturation: As the window length increases, the model’s accuracy improves, reflecting the benefit of capturing longer temporal dependencies in the RRI signal. However, the gain in val_acc from L = 80 to L = 100 is marginal (0.06%), indicating diminishing returns.
- Computational Cost: Longer window lengths require a greater number of recurrent operations per inference run. This directly translates to higher Inference Latency and Energy Consumption.
- Memory Footprint: The memory required to store the LSTM hidden states and process the input buffer scales linearly with the window length (L). For L ≥ 60, the model approaches the available RAM ceiling of the STM32L475VGT6 after accounting for the operating system and peripheral drivers.

**Table A1:** Performance vs. RRI window length.

RRI Window Length	Validation Accuracy	Validation Loss
(L in Beats)	(Val_ACC in %)	(Val_Loss)
20	97.57	0.0770
40	98.00	0.0590
60	98.23	0.0504
80	98.58	0.0416
100	98.64	0.0401

The 40-beat window was ultimately chosen because it delivers a competitive val_acc of 98.00%, while maintaining a significantly lower memory and computational footprint compared to the longer windows. This choice maximises the energy efficiency-to-accuracy ratio necessary for continuous long-term wearable monitoring.
